# Perceived stress in first year medical students - associations with personal resources and emotional distress

**DOI:** 10.1186/s12909-016-0841-8

**Published:** 2017-01-06

**Authors:** Ines Heinen, Monika Bullinger, Rüya-Daniela Kocalevent

**Affiliations:** Department of Medical Psychology, University Medical Center Hamburg-Eppendorf, Hamburg, 20246 Germany

**Keywords:** Stress, First year medical students, Depression, Anxiety, Self-efficacy, Optimism, Coping, Joy

## Abstract

**Background:**

Medical students have been found to report high levels of perceived stress, yet there is a lack of theoretical frameworks examining possible reasons. This cross-sectional study examines correlates of perceived stress in medical students on the basis of a conceptual stress model originally developed for and applied to the general population. The aim was to identify via structural equation modeling the associations between perceived stress and emotional distress (anxiety and depression), taking into account the activation of personal resources (optimism, self-efficacy and resilient coping).

**Methods:**

Within this cross-sectional study, 321 first year medical students (age 22 ± 4 years, 39.3% men) completed the Perceived Stress Questionnaire (PSQ-20), the Self-Efficacy Optimism Scale (SWOP) and the Brief Resilient Coping Scale (BRCS) as well as the Patient Health Questionnaire (PHQ-4). The statistical analyses used *t*-tests, ANOVA, Spearman Rho correlation and multiple regression analysis as well as structural equation modeling.

**Results:**

Medical students reported higher levels of perceived stress and higher levels of anxiety and depression than reference samples. No statistically significant differences in stress levels were found within the sample according to gender, migration background or employment status. Students reported more self-efficacy, optimism, and resilient coping and higher emotional distress compared to validation samples and results in other studies. Structural equation analysis revealed a satisfactory fit between empirical data and the proposed stress model indicating that personal resources modulated perceived stress, which in turn had an impact on emotional distress.

**Conclusions:**

Medical students’ perceived stress and emotional distress levels are generally high, with personal resources acting as a buffer, thus supporting the population-based general stress model. Results suggest providing individual interventions for those students, who need support in dealing with the challenges of the medical curriculum as well as addressing structural determinants of student stress such as course load and timing of exams.

## Background

Stress can be defined as “a condition or feeling experienced when a person perceives that the demands placed on them exceed the resources the individual has available” [1]. Stress can therefore be understood as a perceived imbalance between the demands encountered in daily living and a person’s capability to respond [2, 3]. Medical students may experience stress when curricular demands exceed their resources to deal with them [4], and they have been reported to suffer from higher perceived stress compared to the general population and students in other academic fields (e.g. [5–9]). Dyrbye and Shanafelt concluded that the high degree of perceived stress faced by medical students requires “A Call to Action” [6]. Perceived stress in these studies was assessed with generic questionnaires such as the Perceived Stress Questionnaire (PSQ, e.g. Kohls et al. [10]) and the Perceived Stress Scale (PSS, e.g. Ludwig et al., [11]) or specific questionnaires for medical students such as the Perceived Medical School Stress (PMSS; e.g. Vitaliano et al. [12], Tyssen et al. [13]).

In addition to the level of perceived stress, international (e.g. [7, 13–16]) and German studies (e.g. [8], [17–19]), report on psychosocial consequences such as increased levels of depression and anxiety as well as burnout and reduced quality of life. Study-related stressors experienced by medical students include high workloads, tight time schedules, dissection of corpses, contact with severely ill, suffering and dying patients, and financial problems, as well as language barriers, communication difficulties and cultural differences especially for international students [7, 20–24]. Moffat et al. [4] examined stress during the first year of medical school and found a significant increase in psychological morbidity as measured by the General Health Questionnaire GHQ-12, a screening instrument to detect psychological disorders in the general population and in primary care [25]. Studies focussing on emotional distress as a consequence of prolonged exposure to stressors [15] found increased anxiety and depression scores in medical students – in comparison to the general population [5, 26–28] – as well as increased psychological impairments as measured by the GHQ-12 [4].

Among the personal resources, optimism and self-efficacy have been investigated as buffers of perceived stress. Higher levels of optimism have been found to be associated with less perceived personal stress levels in the general population and in students [29, 30]. In an overview Conversano et al. ([31], p. 25) stated that “optimism may significantly influence mental and physical well-being by the promotion of a healthy lifestyle as well as by adaptive behaviours and cognitive responses, associated with greater flexibility, problem-solving capacity and a more efficient elaboration of negative information”. Studies with adolescent high school students revealed a correlation between higher self-efficacy and higher mental health status on one hand as well as less perceived stress on the other hand [32]. In college students, higher self-efficacy resulted into better mental and physical wellbeing [33, 34]. In medical students, optimism was also associated with higher scores on psychological wellbeing [30] and during the first year of medical education joy as a positive mood decreased while depression increased [35]. Other studies investigated the use of coping strategies by medical students and found that medical students in general use active coping strategies in order to deal with stress experienced within the first year at medical school [4].

During over 40 years of research, evidence accumulates that medical students report a high level of perceived stress and apply individual approaches to cope with it, also by investigating the effects of interventions such as stress reduction trainings [27], peer support programs [36], student focused curricula [37] or wellness courses [11]. Many studies, however, focused on single aspects of the stress experienced by medical students, such as stressors, amount of perceived stress, emotional distress or coping strategies, but failed to investigate conjointly relevant aspects within a general stress model [15, 26]. It is expected that such an approach could contribute to a better understanding of how stress develops in medical students, how the stress experience in medical students differs from that of the general population, how adaptive resources contribute to stress regulation, in medical students and how the stress experience can be addressed via targeted interventions.

Theoretical models unravelling determinants and consequences of stress in medical students have been published by Dyrbye et al. [14], Dunn et al. [38] and Mavor et al. [39]. In 2005, Dyrbye et al. [14] proposed a literature-based interactive model of student-perceived stress, that includes personal factors (e.g. personality traits or coping strategies) and factors related to medical training (e.g. workload, curriculum, ethical conflicts) as hypothetical determinants of student distress. Potential personal (e.g. break ups in relationships, substance abuse or suicide) and professional events (e.g. impaired academic performance, decline in empathy or medical errors) as well as their interaction are viewed as determinants and consequences of the stress level. In 2008, Dunn et al. [38] presented a conceptual model of medical students’ well-being, based on literature review, entitled as the “coping reservoir”. The authors claimed that the coping reservoir changes in a dynamic process, leading to the possible outcomes of either burnout and stress or enhanced resilience and mental health. Personal traits, temperament and coping style have an impact on the coping reservoir by forming the internal structure of the reservoir. The authors proposed that negative input (stress, internal conflicts, time and energy demands) as well as positive input (psychological support, social activities, mentorship and intellectual stimulation) influence students’ personal coping reservoir and may affect either burnout or resilience. In 2014, Mavor et al. [39] proposed a model incorporating interactive effects of self-complexity with group identity and norms. According to this approach, a low level of self-complexity due to the demands of the medical training is unhelpful in buffering stressful situations. Furthermore, a strong group identity (as medical students) can be supportive but can also result in high pressure to follow maladaptive group norms. None of the three models described above been tested empirically so far.

Recently Kocalvent et al. [29] proposed a generalized stress model including self-regulation resources, perceived stress level and its consequences on emotional distress such as anxiety or depression and examined it in the general German population. This model is based on Lazarus and Folkman’s transactional model of stress [2], which postulates that the stress experience is modulated in a dynamic process by appraisal and coping, in that individuals who perceive stress examine and use their presently available coping resources [29]. Lazarus and Folkman’s model [2] contributed to the understanding of stress by identifying direct effects of resources on the perception of stress and indirect effects on stress reactions and broader health-related outcomes [29]. In their population study, Kocalevent et al. [29] operationalized the latent variable resources by self-efficacy, optimism, and joy and the latent variable stress perception by demands, tension and worries. In line with the stress coping model, the results showed that resources explained major part of the variance of stress perception and thus contributed to a transactional understanding of stress by identifying direct effects of resources on stress perception and indirect effects of stress perception on physical and mental fatigue respectively.

**Fig. 1:**
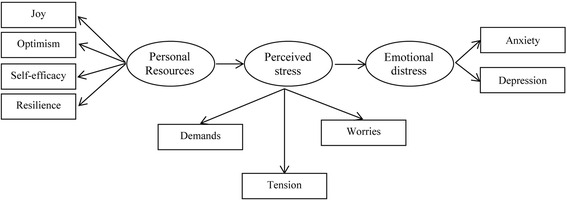
Modified general stress model based on Kocalevent et al. [29]

The main objective of the present study was to investigate empirically on the basis of a transactional conceptual model the stress experience of medical students and its determinants, with the intention of identifying potential interventions to improve student wellbeing in the future. In addition, a theoretical contribution was intended by examining if a modified transactional model of stress originally developed for and tested in the German general population ([29]; see Fig. 1), was applicable to a medical student population. Therefore, we examined the associations between personal resources (specifically self-efficacy, optimism, resilient coping, and joy) and perceived stress (specifically tension, worries, and demands), as well as between perceived stress and emotional distress (specifically depression and anxiety) via structural equation modelling in a cohort of medical students.

In accordance with previous studies, we expected to find higher levels of perceived stress and emotional distress among medical students as compared to the general population and to nonmedical students (e.g. [8, 17, 18]). We also expected to find higher levels of perceived stress, emotional distress and fewer personal resources in female as compared to male students [17, 20, 21], in students with migration background as compared to German students [20] and in students who work part time as compared to students who do not work [7, 20, 21]. We were interested in the effect of personal resources on perceived stress and the effect of perceived stress on emotional distress in medical students as compared to the general population in order to examine the generalizability of the population-based stress model and the benefit of such a general model in a student population.

Finally, we were interested in identifying approaches for interventions to reduce the perceived stress level of medical students either by addressing structural-environmental or individual-regulatory resources. In general, approaches for interventions to reduce the perceived stress level of the medical students and its consequences such as anxiety or depression can address the institutional level or environmental factors of the curriculum – e.g. team based learning or a shorter preclinical phase – [40] or can address the students’ personal resources – e.g. enhancing coping strategies or the perceived self-efficacy.

## Methods

The aim of this study was to test the applicability of a general population-based stress model in a sample of medical students. The effect of personal resources on perceived stress and the effect of perceived stress on emotional distress were examined using a structural equation model (SEM). Figure 1 (see above) shows the underlying theoretical model with latent variables used to represent the respective constructs, namely personal resources, perceived stress, and emotional distress.

### Subjects

The cross-sectional study was conducted at the Medical Faculty of the University of Hamburg, Germany, which in 2012 had introduced an integrative medical curriculum combining preclinical and clinical training based on problem-oriented learning (iMED). The structure of the iMED curriculum includes modular organization of topics presented in a learning loop of increasing complexity from semester 1 to 10 and each semester presents two thematically different modules [39]. All 385 first year medical students of the iMED curriculum (cohort admitted in fall 2013) were asked to complete and return a questionnaire as a learning experience during regular seminars in January 2014, at the beginning of the second, six-week module, and before the written semester exam in February 2014. The total questionnaire consisted of 70 item, containing standardized instruments and single questions about interest in stress reduction courses and counselling, and was completed in 10–15 min. For the analyses presented in this paper subscales of standardized instruments (totalling 42 items) were used. The questionnaires were distributed and collected within an obligatory 120 min seminar on stress and health in 20 groups with up to 20 students each. No incentive was given to the students to participate in the survey, as completion was part of the learning experience regarding stress perception. The seminars were mandatory with an average attendance rate of about 95% of all students. The survey was explained to the students at the end of the first part of the seminar. The questionnaires were distributed to be filled out during the break before the second part of the seminar.

A total of 321 questionnaires could be used for the analyses, yielding a response rate of 83%. The percentage of women (60%) in the sample of 321 students was representative for German medical students in their first year: In fall 2013, 9.381 students started studying medicine at all German medical schools and 5.838 (62.2%) of them were women [41]. The study was conducted according to the principles expressed in the Declaration of Helsinki. Written informed consent was obtained from the participants, data was collected and processed anonymously and decline of participation was possible anytime without any consequences. The dean of the University Medical Center Hamburg-Eppendorf approved the study protocol and the intra-faculty Commission on Teaching and Learning provided consensus.

### Instruments and variables

For this study, the results of four instruments measuring self-efficacy and optimism (SWOP [42]), resilient coping (BRCS [43]), perceived stress (PSQ-20 [44]) and anxiety and depression (PHQ-4, [45]) were chosen for analyses, as the scales of these standardized instruments build the latent variables personal resources, perceived stress and emotional distress (see also below). The scales were selected in accordance to the population-based stress model and are presented in accordance with the structure of the stress model tested of Kocalevent et al. [29]. In addition to the standardized instruments, four sociodemographic questions were included concerning age, gender, part time job and family background. An additional item asked for the students’ approval that the anonymized data of the questionnaire might be used for scientific analyses and publication.

The instruments’ reliability in terms of internal consistency in the current student population was calculated via Cronbach’s alpha [46, 47] and McDonald’s omega (ω_t_) [48]. Cronbach’s alpha is widely used, but also criticized in psychological research [49]. McDonald’s omega is an alternative measure for internal consistency reliability, which is calculated using a 95% confidence interval achieved by bootstrapping [49].

#### Personal resources

The latent variable personal resources was operationalized by the subscales optimism, self-efficacy, joy and resilient coping, assessed with three different standardized instruments.

Optimism and self-efficacy were measured using the Self-Efficacy, Optimism and Pessimism Instrument (SWOP [42]), which is a combination of the self-efficacy questionnaire by Schwarzer and Jerusalem [50] and the optimism questionnaire by Scheier and Carver [51]. The SWOP was validated in a German sample [42] and has been frequently used (e.g. [29, 52–54] and is available as a 9-item short version (K9) comprising of nine items; five items form the self-efficacy scale and two items each measure optimism and pessimism. The SWOP questionnaire uses a four-point response scale ranging from 1: “not correct” to 4: “absolutely correct”. For the optimism scale Cronbach’s α (.72) and McDonald’s ω_t_ (.72 [CI: .63–.78]) showed comparable and good internal consistency, whereas coefficients were weaker for the self-efficacy scale (α: .60; ω_t:_ .01 [CI: 3.5e^−07^ - .10]) and the pessimism scale (α: .47; ω_t:_ .01 [CI: 1.3e^−07^ - .51]) – especially in matters of McDonald’s ω_t_. The pessimism subscale was not used in the multiple regressions and the SEM, but it was used to compare the SWOP score of the medical students in our study with other samples. Joy was measured using the joy scale of the Perceived Stress Questionnaire in its 20 item version (PSQ-20, see below) [50]. The items of the joy scale are answered on a four point rating scale ranging from 1: “almost never” to 4: “usually”. Cronbach’s α (.80) as well as McDonald’s ω_t_ (.81 [CI: .77–.84]) indicated high internal consistency of the joy-scale.

Resilient coping was measured using the Brief Resilient Coping Scale (BRCS), a 4-item questionnaire measuring adaptive – i.e. resilient – coping with stress [43]. Response options are provided on a five point rating scale ranging from 1: “does not describe you at all” to 5: “describes you very well”. The BRCS, which has been translated, validated and normed in Germany [55] and other countries [56], screens for an active and effective problem-solving coping pattern and is considered to be sufficiently valid and reliable [56]. The resilience of medical students has been explored in various studies using different instruments (e.g. [57–60]) and recently in Spain with the BRCS [61]. In our sample Cronbach’s α (.43) as well as McDonald’s ω_t_ (.48 [CI: .37–.55]) showed suboptimal results for the BRCS.

#### Perceived stress

Perceived stress was measured using the subscales worries, tension, and demands of the PSQ-20 [44], following the approach of Kocalevent et al. [29]. All items are answered on a four point rating scale ranging from 1: “almost never” to 4: “usually”. A linear transformation changes the subscale scores to values from 0 to 1. In order to compare the prevalence of perceived stress among medical students to reference groups, the total PSQ-20 score comprised of all four scales was used: joy, worries, tension, and demands. The PSQ-20 was validated in different German adult samples [44] and was previously used in medical and dental students [10, 44, 62–64]. Internal consistency coefficients were high both in terms of Cronbach’s α (worries: .76, tension: .77, demands: .81) as well as McDonald’s ω_t_ (worries: .76 [CI: .71–.80], tension: .77 [CI: .72–.81], demands: .82 [CI: .78–.85]).

#### Emotional distress

Emotional distress was measured with the Patient Health Questionnaire (PHQ-4, [45]) which consists of four items derived from the PHQ-9 [65]. Two items screen for depression by focussing on the two core DSM-IV items for major depressive disorder (PHQ-2), and two more items screen for anxiety, representing the two core DSM-IV items for generalized anxiety disorder by using the first two items of the GAD-7 [65]. Responses are given on a four point rating scale ranging from 0: “not at all” to 3: “nearly every day”. The PHQ-4 is a valid and reliable brief instrument to screen for depression and anxiety and was previously tested in the general German population [66]. According to Kroenke et al. [45], persons scoring above three points or greater in the PHQ-2 or GAD-2 should be examined for clinical relevant depression or anxiety disorder. The PHQ-9 instrument has been used in various surveys with medical students [67–70]. In our sample, Cronbach’s α (PHQ-4: .76, PHQ-2: .62, GAD-2: .70) as well as McDonald’s ω_t_ (PHQ-4: .77 [CI: .72–.82], PHQ-2: .62 [CI: .52–.71], GAD-2: .70 [CI: .59–.77]) showed good results, indicating a satisfactory internal consistency of the instruments.

### Statistical analyses

Means and standard deviations of the subscales used in the present study were calculated at first for the total sample, then separately for subgroups composed of the sociodemographic variables gender, migration background and part time work. Differences between two groups were examined by *t*-tests for independent groups. Differences between more than two groups were calculated using ANOVA. If existing, population norms on personal resources, perceived stress, and emotional distress were inspected for statistical significant differences to the student sample means by one sample *t*-test. Since the sociodemographic variables age, gender, migration background and part time job have been found to be accountable for differences in the stress experience of medical students in previous studies ([7, 17, 20, 21]), they were examined in order to subsequently statistically control them in the stress model if necessary.

Spearman’s rho correlation coefficients and stepwise multiple regression analyses were used to explore the relationships between subscales as components of the stress model. The multiple regressions were also calculated to identify relevant indicator variables for the SEM model. Structural equation modeling (SEM) was performed to test the model fit of the student data set with the postulated general stress model and to examine the relationship of the latent variables by path analysis. The linear structural regression models were carried out with the maximum likelihood method. To assess a SEM model several fit criteria can be used (e.g. [71–74]). Fit indices of the SEM in this study were reported according to Kriston [74] in terms of Discrepancy Test (*χ* ^2^ / df), Standardized Root Mean Residual (SRMR), Root Mean Square Error of Approximation (RMSEA), Tucker-Lewis Index (TLI), Comparative Fit Index (CFI), Normed *χ* ^2^, and Akaike Information Criterion (AIC).

Data were analysed with SPSS/PASW (version 18.0), AMOS (version 22) for the SEM and R (version 3.1.1) for McDonald’s omega (ω_t_). In order to ensure data quality, the total data set was entered twice and inconsistencies between the two data sets were corrected.

## Results

In January 2014, all 385 first year medical students admitted in autumn 2013, were invited to participate in the study, of which 360 students completed the questionnaire. Due to missing values, the final sample included 321 subjects yielding an 83% response rate of all first year medical students of the year 2013 cohort at University Medical Center Hamburg-Eppendorf (60% women, mean age 22 years, 30 with migration background). Table 1 shows the sociodemographic characteristics of the sample.

**Table 1:** Sociodemographic data of the medical students; means and standard deviations (SD) of the variables

	Total *N* = 321, 100%	Male *n* = 127, 39.6%	Female *n* = 194, 60.4%	PSQ-20 M (S.D.)	PHQ-4 M (SD)	PHQ-2 M (SD)	GAD-2 M (SD)
Age, M (S.D.)	21.80 (3.93)	21.79 (3.96)	21.80 (3.93)	Total: .397 (0.154)	Total:2.65 (2.197)	Total:1.26 (1.123)	Total: 1.40 (1.356)
				Male: .383 (.162)	Male: 2.65 (2.136)	Male: 1.36 (1.092)	Male: 1.46 (1.418)
				Female: .407 (.148)	Female: 2.65 (2.242)	Female: 1.36 (1.166)	Female: 1.29 (1.254)
German family background	226 (70.4%)	86 (68.3.1%)	140 (71.8%)	.390 (.151)	2.61 (2.207)	1.24 (1.169)	1.37 (1.307)
International family background	95 (29.6%)	40 (31.7%)	55 (28.2%)	.412 (.160)	2.76 (2.182)	1.29 (1.009)	1.46 (1.472)
European (EU) family background	28 (8.7%)	10 (7.9%)	18 (9.2%)	.378 (.138)	2.29 (1.740)	1.14 (.932)	1.14 (1.208)
Family background from outside EU	41 (12.8%)	22 (17.5%)	19 (9.7%)	.402 (.167)	2.78 (2.361)	1.29 (1.055)	1.49 (1.583)
Internationally mixed family background	26 (8.1%)	8 (6.3%)	18 (9.2%)	.462 (.163)	3.23 (2.179)	1.46 (1.029)	1.77 (1.531)
No part time job	240 (74.8%)	94 (74.6%)	146 (74.9%)	.394 (.153)	2.74 (2.256)	1.27 (1.123)	1.47 (1.396)
Part time job	81 (25.2%)	32 (25.4%)	49 (25.2%)	.407 (.159)	2.41 (2.005)	1.22 (1.129)	1.19 (1.396)
Up to 10 h per week	63 (19.6%)	19 (15.1%)	44 (22.6%)	.394 (.155)	2.25 (1.805)	1.16 (1.096)	1.10 (1.073)
More than 10 h per week	18 (5.6%)	13 (10.3%)	5 (2.6%)	.453 (.167)	2.94 (2.578)	1.44 (1.247)	1.50 (1.618)

The following results are presented according to the latent variables in the general stress model by Kocalevent et al. ([29], see Fig. 1), representing the sequence from personal resources over perceived stress to emotional distress.

### Personal resources in medical students

The mean self-efficacy score of the SWOP was 2.87 (SD = 0.39; range: 1–4) and the mean of the optimism-scale of the SWOP was 3.00 (SD = 0.67; range: 1–4). As compared to the 726 subjects of the SWOP clinical validation sample of patients with psychosomatic (*n* = 171) and somatic conditions (*n* = 555, diabetes, skin disease, slipped disk, transplant [42]), the medical students in our study scored statistically significantly higher in self-efficacy (students: *M* = 2.87, SD = .39; validation sample: *M* = 2.81, SD = 0.62; *p* = .004) and optimism (students: *M* = 3.00, SD = .67; validation sample: *M* = 2.84, SD = 0.88; *p* < .001) and significantly lower in pessimism (students: *M* = 1.93, SD = .59; validation sample: *M* = 2.22, SD = 0.79; *p* < .001, [42]). In comparison to German surgeons in a study of Mache et al. [54] the students in our sample showed significantly lower self-efficacy (students: *M* = 2.87, SD = .39; surgeons: *M* = 3.23, SD = 0.71, *p* < .001), lower optimism (students: *M* = 3.00, SD = .67; surgeons: *M* = 3.57, SD = 0.67; *p* < .001) and higher pessimism scores (students: *M* = 1.93, SD = .59; surgeons: *M* = 1.82, SD = 0.79; *p* < .001, [48]) than the surgeons.

The mean in the BRCS-scale, measuring resilient coping, was 15.28 (SD = 2.10; range: 9–20) and significantly higher than the scores of the 140 subjects of the validation sample (individuals with rheumatoid arthritis, *M* = 14.56, SD = 1.01, *p* < .001; [43]). The mean in our sample was also significantly higher than the BRCS scores in a Spanish psychology student sample studied by Limonero et al. (*M* = 14.90, SD = .91, *p* = .001; [61]).

### Perceived stress in medical students

The overall mean of the perceived stress level measured by the PSQ-20 was 0.40 (SD = 0.15; range: .02–.82). The perceived stress level of the medical students in our sample was significantly higher than the level of the age related German norm population (*M* = .30, SD = 0.15; T(320) = 11.290, *p* < .001; [75]) and significantly higher than the stress level of 249 German second year medical students (*M* = 0.37, SD = .17; T(320) = 3.146, *p* = .002) in a sample studied by Fliege et al. [64]. The students’ scores were categorized into three groups according to a study of Bergdahl and Bergdahl [76] and Kocalevent et al. [75]:

- ▶ Group 1: ≤ mean (M) + 1 standard derivation (SD) = mean stress level
- ▶ Group 2: (> M + 1 SD) – (≤ M + 2 SD) = slightly increased stress level
- ▶ Group 3: > M + 2 SD = high stress level

In our study 269 (83.8%) students sorted into group 1 showed a mean stress level, 41 (12.8%) students sorted in group 2 showed a slightly increased stress level and 11 (3.4%) students sorted in group 3 showed a high stress level. When these three groups were formed on the basis of the German validation sample’s results (*M* = .30, SD = .15, [44]), only 210 (65.4%) of the students in our study showed the mean stress level (group 1 of the German validation sample), 78 (24.3%) students showed slightly increased stress levels (group 2 of the German validation sample) and 33 (10.3%) students showed high stress levels (group 3 of the German validation sample).

The perceived stress level measured by the PSQ-20 was analysed for variability within subgroups of medical students. There were no statistically significant differences according to gender (T(319) = 1.366, *p* = .173) or migration background (*F* = 1.843, p = .139). Furthermore perceived stress did not differ in students with a part-time job as compared to those without (T(319) = −.672, *p* = .502). There was no difference in perceived stress between students who do not work (M = .39), students who work up to ten hours (*M* = .39) and students who work 11 h or more (*M* = .45; *F* = 1.251, *p* = .288).

### Emotional distress in medical students

The PHQ-4 screening measure for depression and anxiety revealed a mean of 2.65 (SD = 2.20; range: 0–12) for the sum score, for the PHQ-2 (screening for depression) a mean of 1.26 (SD = 1.12; range: 0–6) and for the GAD-2 (screening for anxiety) a mean of 1.40 (SD = 1.36; range: 0–6). In addition to the numerical scoring, the PHQ-2 and the GAD-2 can be interpreted categorically, using severity scores [45]. In the PHQ-2 (screening for depression) 37 (11.5%) students had a score of three points or higher, in the GAD-2 (screening for anxiety) 59 (18.4%) students scored three points or higher. According to Kroenke et al. [45] persons scoring three points and higher should be further evaluated.

There were no statistically significant differences between male and female students in the PHQ-4 (T(319) = .004, *p* = .997), the PHQ-2 (T(319) = −1.339, *p* = .181) and the GAD-2 (T(319) = 1.115, *p* = .266). There were no significant differences between students with different migration background in the PHQ-4 (*F* = .934, *p* = .425), PHQ-2 (*F* = .406, *p* = .749) and GAD-2 (*F* = 1.078, *p* = .358), but students with international family background scored higher in the PHQ-2 and GAD-2 than the students with German family background. Differences between working (more or less than ten hours per week) and non-working students in the PHQ-4 (*F* = 1.378, *p* = .254), PHQ-2 (*F* = .508, *p* = .602) and GAD-2 (*F* = 1.939, *p* = .146) were non-significant.

Within the PHQ-2, screening for depression, medical students showed a significantly higher mean (*M* = 1.26, SD = 1.12) than the general population (*M* = .94; T(320) = 5.083, *p* < .001; [66]) and a percentile about 70 of the students in comparison to the general population. Again, in comparison to the age-matched norm population (14–24 years, *M* = .83, SD = 1.11) our student sample scored significantly higher (T(320) = 6.838, *p* < .001).

Within the GAD-2, screening for anxiety, (students: *M* = 1.40, SD = 1.36; German population: *M* = .82; T(320) = 7.603, *p* < .001; [66]) a similar result was found, indicating that the students score on a 70 percentile, too. As before, in comparison to the age-matched norm population (14–24 years, *M* = .72, SD = 1.08) our student sample scored significantly higher (T(320) = 8.924, *p* < .001).

### Correlations

Within the different measures of personal resources, joy showed the highest correlations with stress and emotional distress (PSQ-20 worries: *r* = −.56, PSQ-20 tension: *r* = -.54, PHQ2 depression: *r* = −.55, GAD2 anxiety: *r* = −.51, PHQ4 *r* = −.60). The perceived stress level assessed by all four PSQ-20 subscales (with the joy scale *included* and *reversed*) correlated highly with the depression (PHQ-2, *r* = .51) and the anxiety (GAD-2, *r* = .57) subscales of the PHQ-4 as well as with the PHQ-4 sum score (*r* = .62). Anxiety assessed by GAD-2 correlated highest with the PSQ-20 worries subscale (*r* = .60; see Table 2).

**Table 2:** Correlations between the scores contributing to the variables personal resources, perceived stress, and emotional distress

Variables	Operatio-nalisation	BRCS sum	SWOP Self-efficacy	SWOP Optimism	SWOP Pessimism	PSQ-20 Joy	PSQ-20 Worries	PSQ-20 Tension	PSQ-20 Demands	PHQ-2 Depression	GAD-2 Anxiety	PHQ-4 (sum)	PSQ-20 (sum)
Personal resources	Resilient coping (BRCS)	1.00	.31^a^	.27^b^	–.15^b^	.24^b^	–.16^b^	–.12^a^	–.10	–.14^b^	–.14^b^	–.15^b^	–.18^b^
	Self-efficacy (SWOP)		1.00	.36^b^	–.27^b^	.39^b^	–.38^b^	–.24^b^	–.16^b^	–.26^b^	–.31^b^	–.32^b^	–.36^b^
	Optimism (SWOP)			1.00	–.29^b^	.58^b^	–.44^b^	–.40^b^	–.20^b^	–.38^b^	–.40^b^	–.44^b^	–.48^b^
	Pessimism (SWOP)				1.00	–.28^b^	.23^b^	.20^b^	0.05	.23^b^	.21^b^	.25^b^	.24^b^
	Joy (PSQ-20)					1.00	–.56^b^	–.54^b^	–.26^b^	–.55^b^	–.51^b^	–.60^b^	–.75^b^
Perceived stress	Worries (PSQ-20)						1.00	.61^b^	.43^b^	.50^b^	.60^b^	.63^b^	.81^b^
	Tension (PSQ-20)							1.00	.58^b^	.42^b^	.48^b^	.51^b^	.86^b^
	Demands (PSQ-20)								1.00	.21^b^	.26^b^	.26^b^	.73^b^
Emotional distress	Depression (PHQ-2)									1.00	.53^b^	.86^b^	.51^b^
	Anxiety (GAD-2)										1.00	.87^b^	.57^b^
	PHQ-4 (sum)											1.00	.62^b^
	PSQ-20 (sum)												1.00

^a^Correlation is statistically significant on a .05 level (two-sided)

^b^Correlation is statistically significant on a .01 level (two-sided)

### Multiple regression of personal resources on perceived stress

To explore the effects of personal resources on perceived stress, three stepwise multiple regressions were calculated with the PSQ-20 subscales (1) worries, (2) tension, and (3) demand as dependent variable. Resilient coping (BRCS), the PSQ-20 subscale joy and the SWOP scales self-efficacy and optimism were used as independent variables, representing personal resources. In the multiple regressions the joy scale was *not* reversed, therefore higher joy scores indicate *more* joy. The variance of worries was mainly explained by joy and self-efficacy (.35 corrected R^2^). With tension as dependent variable, joy and optimism explained 32% of the variance (.32 corrected R^2^). With the PSQ-20 subscale demands as dependent variable, joy was the only relevant variable, explaining .06 corrected R^2^ (see Table 3).

**Table 3:** Stepwise multiple regressions with worries, tension and demands as dependent variable

Dependent variable	Independent variables	B (95% CI_l_–CI_u_)^a^	SE	ß	*p*	Corrected R^2^
Worries (PSQ-20)	Joy (PSQ-20)	–.48 (–.57–−.39)	.05	–.51	<.001	.35
	Self-efficacy (SWOP)	–.08 (−.12–−.03)	.02	–.16	.001	
Tension (PSQ-20)	Joy (PSQ-20)	–.47 (−.57–−.36)	.05	–.48	<.001	.32
	Optimism (SWOP)	–.04 (−.07–−.01)	.02	–.14	<.014	
Demands (PSQ-20)	Joy (PSQ-20)	–.27 (−.38–−.15)	.06	–.25	<.001	.06

^a^B with lower (CI_l_) and upper limit (CI_u_) of the 95%-confidence interval

### Multiple regressions on emotional distress

In order to explore the effects of perceived stress on emotional distress, three stepwise multiple regressions were performed. Dependent variables were (1) PHQ-4, (2) PHQ-2 and (3) GAD-2, independent variables were the PSQ-20 subscales worries, tension and demands. With the PHQ-4 as dependent variable, the worries, tension and demands explained .45 corrected R^2^. With the PHQ-2 (screener for depression) as dependent variable, the PSQ-20 subscales worries, tension and demands explained .29 corrected R^2^. And with the GAD-2 (screener for anxiety) as dependent variable, the PSQ-20 subscales worries, tension and demands explained .41 corrected R^2^ (see Table 4).

**Table 4:** Stepwise multiple regressions with PHQ-4, PHQ-2 and GAD-2 as dependent variable

Dependent variable	Independent variables	B (95% CI_l_–CI_u_)^a^	SE	ß	*p*	Corrected R^2^
PHQ-4 (sum)	Worries (PSQ-20)	6.02 (4.77–7.26)	.63	.51	<.001	.45
	Tension (PSQ-20)	3.37 (2.07–4.67)	.66	.29	<.001	
	Demands (PSQ-20)	–1.13 (−2.16–−.11)	.52	–.11	.030	
PHQ-2 (Depression)	Worries (PSQ-20)	2.34 (1.62–3.06)	.37	.35	<.001	.29
	Tension (PSQ-20)	1.65 (.90–2.40)	.38	.28	<.001	
	Demands (PSQ-20)	–.68 (−1.27–−.08)	.30	–.13	.026	
GAD-2 (Anxiety)	Worries (PSQ-20)	3.60 (2.81–4.39)	.40	.59	<.001	.41
	Tension (PSQ-20)	1.49 (.73–2.25)	.39	.21	<.001	

^a^B with lower (CI_l_) and upper limit (CI_u_) of the 95%-confidence interval

### Structural equation modeling

**Fig. 2:**
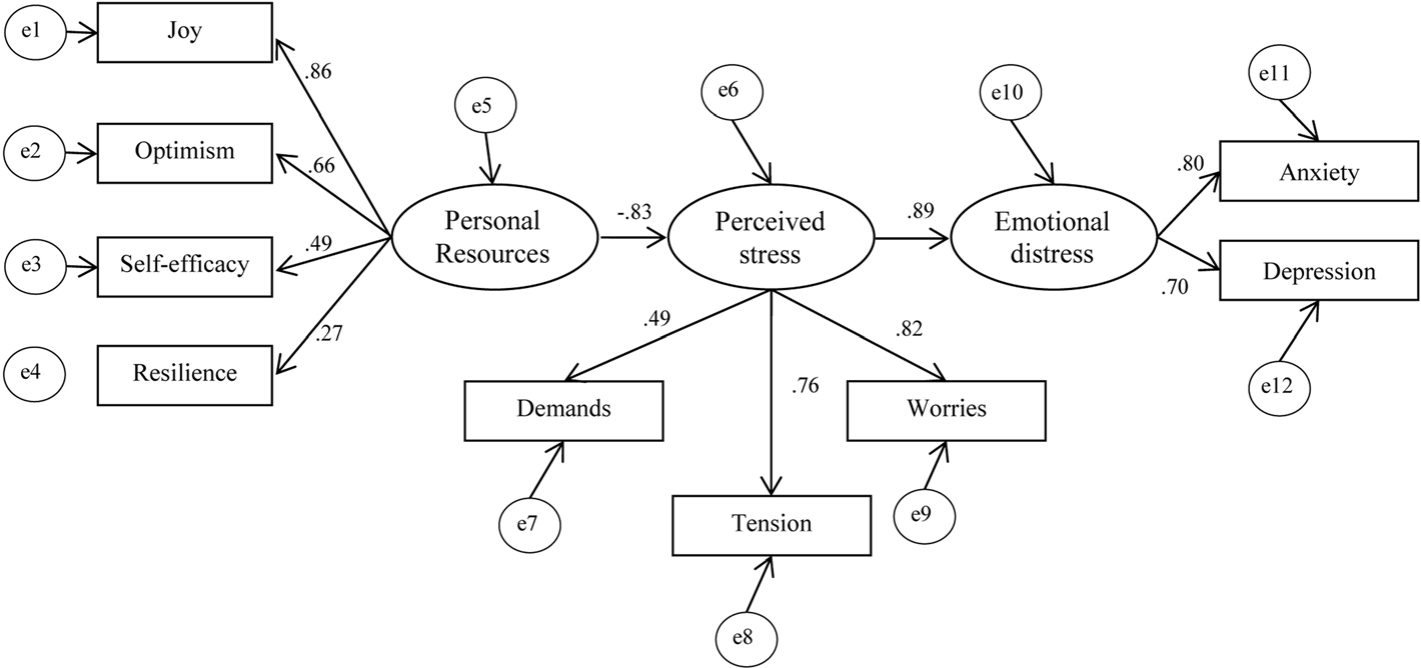
Modified transactional stress model including personal resources, perceived stress and emotional distress. Legend: Circles: unobserved residual variables with fixed regression weight of 1; rectangles: observed indicator variables; ovals: unobserved latent variables; numbers at lines with arrows at each end are correlation coefficients; numbers at lines with arrows at one end are squared regression coefficients; no constraints parameters. Fit Indices: Discrepancy test (*χ* ^2^ / df) =112.304/25, *p* < .001, SRMR = .0595, RMSEA = .104, CFI = .915

Structural equation modeling was used to examine if the population based stress model [29] can be applied to a sample of medical students (see Fig. 2). The latent variable personal resources was operationalised by joy (*not* reversed subscale of the PSQ-20), resilience (BRCS), optimism and self-efficacy (SWOP subscales). The latent variable perceived stress was operationalised by demands, tension and worries (PSQ-20). The latent variable emotional distress was operationalised by depression (PHQ-2) and anxiety (GAD-2). Since the sample was homogeneous in age and did not differ in the latent variables by gender, migration background or part time job we did not control for these variables in the SEM.

**Fig. 3:**
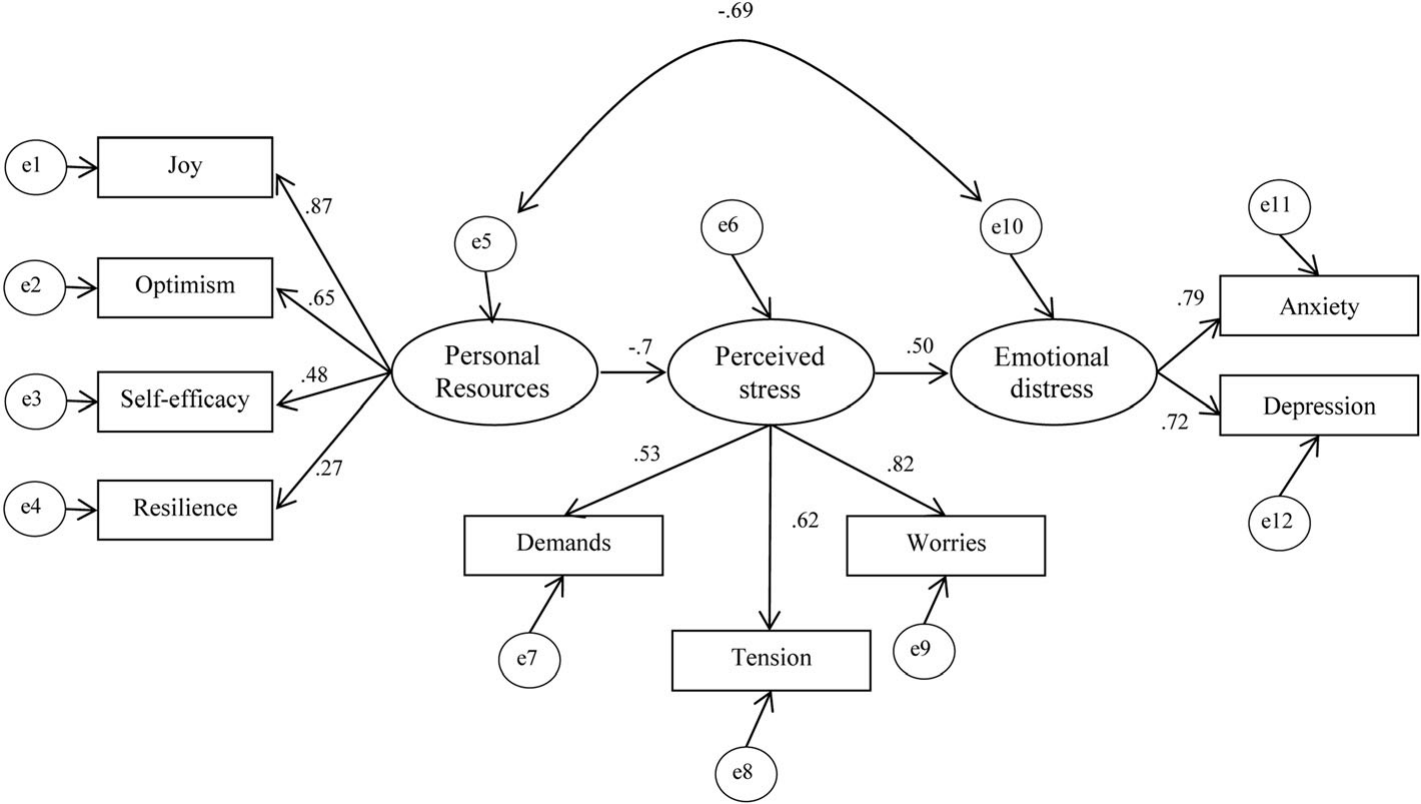
Modified transactional stress model with a direct path between personal resources and emotional distress. Legend: Circles, unobserved residual variables with fixed regression weight of 1; rectangles, observed indicator variables; oval, unobserved latent variables; numbers at lines with arrows at each end are correlation coefficients; numbers at lines with arrows at one end are squared regression coefficients; no constraints parameters. Fit Indices: Discrepancy test (*χ* ^2^ / df) =98.261/24, *p* < .001, SRMR = .0562, RMSEA = .098, CFI = .928

Results of model testing showed the discrepancy *χ* ^2^test to be statistically significant (*χ* ^2^ = 112.304, df = 25, *p* < .001), indicating a difference between theoretical and observed variable. The normed *χ* ^2^ test (4.49) was below the acceptance level of 5. The CFI (.915) was above the threshold, the RMSEA (.104) and SRMR (.0595) were acceptably low and the TLI (.878) was very close to the acceptance level. The Akaike Information Criterion (AIC) was 170.304 (see Table 5). In order to optimize the model fit a second SEM analysis, according to the general stress model [29], was performed with the above latent variables, however allowing a direct path between personal resources and emotional distress (see Fig. 3).

In the second SEM analysis, the TLI (.892) closely approached to the acceptance level of .90 and the CFI (.928) exceeded .90. The RMSEA (0.098 with .079–.119 confidence interval) was satisfactory, supporting the acceptance of the model. The discrepancy *χ* ^2^ test was lower, but still significant (*χ* ^2^ = 98.261, df = 24, *p* < .001), while the normed *χ* ^2^ test was 4.09 and therefore supported the acceptability of the model. The SRMR (.0562) was below the acceptance level of .08 and the model had an AIC of 158.261. Since lower values of the AIC are more preferable, the second model with the direct path between personal resources and emotional distress is preferable. Never the less the quality of the fit indices indicate that further modification could enhance the model fit. All fit indices and their acceptance levels following Kriston et al. ([74]) are shown in Table 5.

**Table 5:** Global goodness-of-fit measures in the two models tested (goodness-of-fit index with recommendations following Kriston et al. [74])

Goodness-of-fit index with recommendations (the most strict recommendation is presented first)	1^st^model, original stress model	2^nd^ model with direct path from personal resources to emotional distress
Discrepancy Test (*χ* ^2^ / df)	112.304/25, *p* < .001	98.261/24, *p* < .001
Standardized Root Mean Residual (SRMR) <0.05, <0.08^a^	. 0595	.0562
Root Mean Square Error of Approximation (RMSEA) <.05, <.06, <.08	.104	.098 (CI .079–.119)
Tucker-Lewis Index (TLI) ≥.95, ≥.90, ≥.80	.878	.892
Comparative Fit Index (CFI) ≥.95, ≥.90, ≥.80	.915	.928
Normed *χ* ^2^ <1.0, <1.5, <2.0, <3.0, <5.0	4.49	4.09
Akaike Information Criterion (model comparisons: smaller value is preferred)	170.304	158.261

^a^The multiple values indicate diverse recommendations with the strictest recommendation as the first value

## Discussion

The present study attempted to contribute to the transactional understanding of stress by testing a general population-based stress model in a cohort of medical students, and examining direct effects of personal resources on perceived stress and indirect effects on emotional distress, operationalised by depression and anxiety levels.

### Personal resources

Personal resources of the medical students were operationalized by the subscales optimism and self-efficacy of the SWOP [42], the subscale joy of the PSQ-20 [44] and resilient coping assessed with the BRCS [43]. The medical students in our study showed increased levels in these four aspects to validation samples ([42, 43]) and population norms [75]. Never the less, the students in our sample showed significantly lower self-efficacy, lower optimism and higher pessimism scores than German surgeons in a study of Mache et al. In contrast, the resilient coping strategies of the students in our sample were higher than in a Spanish psychology student sample [61]. The above comparisons provide an indication about the level of resources. Even though the students in our study had very high levels in optimism, self-efficacy, joy and resilient coping, they experienced high levels of stress.

Personal resources (joy and optimism in particular) were related to students’ stress perception in that high personal resources reduced perceived stress. These empirical findings are in line with the “coping reservoir” postulated by Dunn et al. [38], which corresponds to personal resources such as self-efficacy and optimism in the model tested.

Within the multiple regressions on perceived stress, joy was the only resource that could explain the variance of the PSQ-20 subscales worries, tension, and demands. To reduce the worries and tension medical students experience it might therefore be particularly helpful to enhance the students’ experience of joy in their medical training. In contrast to the general stress-model tested by Kocalevent et al. [29], the latent variable personal resources included resilient coping, as resilience and coping styles have been found to be a core personal competency of medical students [77] and to be important for their stress perception [78]. Although, coping strategies are an essential part of the transactional stress model by Folkman and Lazarus [2], the resilient coping strategies measured by the BRCS were not an important personal resource in our study. One reason might be the high mean of this scale, indicating a ceiling effect of the BRCS score. Another reason might be the ultra-brief BRCS scale itself, which might not be able to detect sufficiently the variance of resilient coping strategies in medical students. In the second SEM, when a direct path between personal resources and emotional distress was added to the model, the effect of perceived stress on emotional distress was considerably reduced. Personal resources – joy and optimism in particular – had a reducing effect on anxiety and depression levels. The fit indices of both SEM models show that our model is not perfectly fitting the data (e.g. the *χ* ^2^ score), even though the second model shows better results than the first one. But there are some shortcomings associated with the *χ* ^2^ test statistics, as it is sensitive to violation of assumptions (e.g. large sample size or normal distribution), model complexity (more parameters lead to a better model fit) and sample size (higher sample size leads to a higher *χ* ^2^ value) [79]. The normed *χ* ^2^ test suggest never the less a reasonable model fit.

### Perceived stress

In line with other studies, the medical students in our study showed higher levels of perceived stress than the age specific German norm population [75]. However, medical students reported also strong personal resources (optimism, self-efficacy, joy and resilient coping strategies), suggesting that the coping efforts were not effective in reducing perceived stress. The students in our study also showed higher stress levels than medical students in their second year examined in 2005 by Fliege et al. [64]. There are at least two possible explanations for this result: First, it is possible that the general level of perceived stress among medical students has increased within the last ten years. Second, it is possible that the first semester is a time, where many personally relevant changes take place within the students’ lives: Examples are leaving home and living on their own for the first time, orientation in a new city away from home, establishing new relationships, and habituation to the processes and examinations at medical school. These findings were in line with the results of earlier studies showing that university students have to deal with stressors like academic and social demands, examination outcome and personal competence [80]. In addition, medical students face training specific stressors such as dissecting corpses [22, 23] and interactions with suffering, chronical ill and dying patients [24]. In contrast to other studies, no statistically significant differences were found between female and male students [17, 21, 81], students with and without migration background [20] and students who work and do not work in addition to their medical training [7, 20, 21]. One reason that higher levels of perceived stress were not found in the subgroups might be the already very high level of stress expressed by the students in our study and therefore the ceiling effect could have prevented further variations to be found within the different subgroups. In addition, the PSQ-20 did not focus on the specific aspects of medical student stress. Therefore, differences between subgroups might have been undetected. Perceived stress was mainly characterized by tension and worries. Higher levels of perceived stress were positively related to higher levels of emotional distress, operationalized by anxiety and depression. At the same time, higher levels of perceived stress were associated with lower levels of joy.

### Emotional distress

The PHQ-4 score – and the PHQ-2 and GAD-2 scores respectively –, indicate the presence of more symptoms of depression and anxiety in the student sample than in the general population and in the age-matched norm population. These results are in line with our expectations and earlier studies [67–69], where the PHQ-9 was used to detect symptoms of depression.

According to the multiple regression analyses, high levels of stress were strongly associated with anxiety. Counselling services or seminars focusing on anxiety might therefore be a useful option to reduce stress within medical education – especially when starting medical school, within the first year of medical education. A recent study by Wild et al. [82] showed the effectiveness of relaxation techniques such as autogenous training and progressive muscle relaxation according to Jacobson (PMR) in reducing burnout and trait and state anxiety levels in medical students.

On the other hand, medical students in our sample showed higher scores in the BRCS – measuring resilient coping strategies – and the optimism and self-efficacy scales of the SWOP than the validation samples and other student groups, which can be interpreted that the medical students already have a higher reservoir on these personal resources than many other people do. Never the less, these personal resources seem not be sufficient to buffer the students’ perceived stress in order to reduce the levels of depression and anxiety. This result supports the notion that interventions on the institutional level or environmental factors of the curriculum [40] or interventions trying to change the general situation of the students might be more successful in reducing the perceived stress level and their consequences such as anxiety or depression than interventions focusing on enhancing students’ personal resources like coping strategies or self-efficacy.

Evidence of higher perceived stress and higher emotional distress however suggest that interventions oriented at strengthening self-regulation could have a potential benefit in the subgroup of medical students with high psychological impairment. So far, stress management in medical students focuses primarily on relaxation and cognitive skills. Deckro et al. [83] examined the effect of a 6-week mind/body intervention on college students’ psychological distress, anxiety, and perception of stress, consisting of group-training sessions in the relaxation response and cognitive behavioral skills. Results indicate significant reductions in psychological distress, state anxiety and perceived stress.

US medical schools offer a student wellness program, according to the regulations of the Liaison Committee on Medical Education (LCME, [84]), in order to support medical students in coping with study related pressures. In the UK, the General Medical Council published a guidance on supporting students with mental health conditions [85] in order to support medical students in coping with stressful situations. To date, comparable official guidelines have not been published in Germany, even though the high levels of stress, anxiety and depression among German medical students have been reported in various studies and were detected in our study as well [8–10, 17, 19, 37, 64].

### Limitations

Limitations of this study pertain to the measurement of stress. It has been much debated whether or not to measure stressors in terms of objective conditions (such as course load) or subjective experience (such as perceived stress). In the present study, results can be interpreted within the framework of an individual’s subjective perception of stress. Furthermore, the study design needs to be acknowledged. We present a cross-sectional study, as longitudinal analysis would have gone beyond the scope of this paper. Longitudinal analysis of the stress model, including personal resources, stress perception, emotional distress, and subjective mental health state among medical students remains to be carried out in future studies.

Finally, the percentage of international students in our sample is higher than in the official statistics of 2013 reporting 9,381 first year medical students in Germany, including 1,912 international students (20.4%; including 1,106 international female students (57.8%), [78]). The difference in international students’ rates might be a result of cosmopolitan Hamburg being preferred over smaller German university cities.

Another limitation refers to the fact that our study included only student of one medical school. The inclusion of different medical school in Germany would result in a broader picture of the levels of perceived stress and emotional distress in medical students.

## Conclusion

The students reported higher levels of perceived stress, anxiety and depression than the general German population validation samples or students in earlier studies. No significant differences between subgroups by gender, migration background and job status were found. We observed that the amount of perceived stress in medical students was buffered by joy, optimism and self-efficacy, and determined levels of anxiety and depression. The students already had higher levels of resilient coping, optimism and self-efficacy than the validation samples, but these personal resources could not prevent the increased levels of perceived stress, anxiety and depression. Interventions on the students’ side – especially supporting their personal resources – may not be seem to be sufficient to lower the perceived stress levels and the levels of anxiety and depression as the students already had high levels of personal resources but never the less experienced much stress. Therefore, we think that it is appropriate to consider other possible interventions on the structural level. Institutional interventions might focus on changes within the medical curriculum or the examination schedule with the intention to lower exposure to academic stressors.

## References

[1] American Institute of Stress. What is Stress? [http://www.stress.org/what-is-stress/] Access Date 26 Aug 2016

[2] Lazarus RS, Folkman S (1984). Stress, appraisal, and coping.

[3] Antonowsky A (1979). Health, stress, and coping.

[4] Moffat KJ, McConnachie A, Ross S, Morrison JM (2004). First year medical student stress and coping in a problem-based learning medical curriculum. Med Educ.

[5] McGuire FL (1966). Psycho-social studies of medical students: a critical review. J Med Educ.

[6] Dyrbye LN, Shanafelt TD (2011). Commentary: medical student distress: a call to action. Acad Med.

[7] Dyrbye LN, Harper W, Durning SJ, Moutier C, Thomas MR, Massie FSJ, Eacker A, Power DV, Szydlo DW, Sloan JA (2011). Patterns of distress in US medical students. Med Teach.

[8] Seliger K, Brähler E (2007). Psychische Gesundheit von Studierenden der Medizin. Psychotherapeut.

[9] Voltmer E, Kotter T, Spahn C (2012). Perceived medical school stress and the development of behavior and experience patterns in German medical students. Med Teach.

[10] Kohls NB, Bussing A, Sauer S, Riess J, Ulrich C, Vetter A, Jurkat HB (2012). Psychological distress in medical students - a comparison of the Universities of Munich and Witten/Herdecke. Z Psychosom Med Psychother.

[11] Ludwig AB, Burton W, Weingarten J, Milan F, Myers DC, Kligler B (2015). Depression and stress amongst undergraduate medical students. BMC Med Educ.

[12] Vitaliano PP, Maiuro RD, Mitchell E, Russo J (1989). Perceived stress in medical school: resistors, persistors, adaptors and maladaptors. Soc Sci Med.

[13] Tyssen R, Vaglum P, Gronvold NT, Ekeberg O (2001). Suicidal ideation among medical students and young physicians: a nationwide and prospective study of prevalence and predictors. J Affect Disord.

[14] Dyrbye LN, Thomas MR, Shanafelt TD (2005). Medical student distress: causes, consequences, and proposed solutions. Mayo Clin Proc.

[15] Dyrbye LN, Thomas MR, Shanafelt TD (2006). Systematic review of depression, anxiety, and other indicators of psychological distress among U.S. and Canadian medical students. Acad Med.

[16] Dyrbye LN, Schwartz A, Downing SM, Szydlo DW, Sloan JA, Shanafelt TD (2011). Efficacy of a brief screening tool to identify medical students in distress. Acad Med.

[17] Jurkat H, Hofer S, Richter L, Cramer M, Vetter A (2011). Quality of life, stress management and health promotion in medical and dental students. A comparative study. Dtsch Med Wochenschr.

[18] Kötter T, Voltmer E (2013). Stressbelastung von Medizinstudierenden messen: Übersetzung des “Perceived Medical School Stress Instruments” in die deutsche Sprache. GMS - Zeitschrift für medizinische Ausbildung.

[19] Jurkat HB, Richter L, Cramer M, Vetter A, Bedau S, Leweke F, Milch W (2011). Depression and stress management in medical students. A comparative study between freshman and advanced medical students. Nervenarzt.

[20] Malau-Aduli BS (2011). Exploring the experiences and coping strategies of international medical students. BMC Med Educ.

[21] Miller GD, Kemmelmeier M, Dupey P (2013). Gender differences in worry during medical school. Med Educ.

[22] Horne DJ, Tiller JW, Eizenberg N, Tashevska M, Biddle N (1990). Reactions of first-year medical students to their initial encounter with a cadaver in the dissecting room. Acad Med.

[23] Madill A, Latchford G (2005). Identity change and the human dissection experience over the first year of medical training. Soc Sci Med.

[24] MacLeod RD, Parkin C, Pullon S, Robertson G (2003). Early clinical exposure to people who are dying: learning to care at the end of life. Med Educ.

[25] Goldberg D (1978). GHQ-12.

[26] Stewart SM, Betson C, Lam TH, Marshall IB, Lee PW, Wong CM (1997). Predicting stress in first year medical students: a longitudinal study. Med Educ.

[27] McGrady A, Brennan J, Lynch D, Whearty K (2012). A wellness program for first year medical students. Appl Psychophysiol Biofeedback.

[28] Stewart SM, Betson C, Marshall I, Wong CM, Lee PW, Lam TH (1995). Stress and vulnerability in medical students. Med Educ.

[29] Kocalevent RD, Klapp BF, Albani C, Brahler E (2013). Zusammenhänge von Ressourcen, chronisch aktiviertem Distress und Erschöpfung in der deutschen Allgemeinbevölkerung [Associations of resources factors, chronic activated distress, and fatigue in the German general population]. Psychother Psychosom Med Psychol.

[30] Krageloh CU, Henning MA, Billington R, Hawken SJ (2015). The relationship between quality of life and spirituality, religiousness, and personal beliefs of medical students. Acad Psychiatry.

[31] Conversano C, Rotondo A, Lensi E, Della Vista O, Arpone F, Reda MA (2010). Optimism and its impact on mental and physical well-being. Clin Pract Epidemiol Ment Health.

[32] Moeini B, Shafii F, Hidarnia A, Babaii GR, Birashk B, Allahverdipour H (2008). Perceived stress, self-efficacy and its relations to psychological well-being status in Iranian male high school students. Soc Behav Pers.

[33] Varghese RP, Norman TSJ, Thavaraj HS (2015). Perceived Stress and Self Efficacy among College Students: A Global Review. Int J Hum Resour Manag Res.

[34] Torres JB, Solberg VS (2001). Role of self-efficacy, stress, social integration, and family support in Latino college student persistence and health. J Vocat Behav.

[35] Wolf TM, von Almen TK, Faucett JM, Randall HM, Franklin FA (1991). Psychosocial changes during the first year of medical school. Med Educ.

[36] Hillis J, Morrison S, Alberici F, Reinholz F, Shun M, Jenkins K (2012). ‘Care Factor’: engaging medical students with their well-being. Med Educ.

[37] Kiessling C, Schubert B, Scheffner D, Burger W (2004). First year medical students’ perceptions of stress and support: a comparison between reformed and traditional track curricula. Med Educ.

[38] Dunn LB, Iglewicz A, Moutier C (2008). A conceptual model of medical student well-being: promoting resilience and preventing burnout. Acad Psychiatry.

[39] Mavor KI, McNeill KG, Anderson K, Kerr A, O’Reilly E, Platow MJ (2014). Beyond prevalence to process: the role of self and identity in medical student well-being. Med Educ.

[40] Slavin SJ, Schindler DL, Chibnall JT (2014). Medical student mental health 3.0: improving student wellness through curricular changes. Acad Med.

[41] Bundesamt S (2014). Studierende an Hochschulen - Vorbericht - Wintersemester 2013/2014. Bildung und Kultur.

[42] Scholler G, Fliege H, Klapp BF (1999). Fragebogen zu Selbstwirksamkeit, Optimismus und Pessimismus: Restrukturierung, Itemselektion und Validierung eines Instrumentes an Untersuchungen klinischer Stichproben. PPmP Psychotherapie Psychosomatik Medizinische Psychologie.

[43] Sinclair VG, Wallston KA (2004). The development and psychometric evaluation of the Brief Resilient Coping Scale. Assessment.

[44] Fliege H, Rose M, Arck P, Levenstein S, Klapp BF (2001). Validierung des “Perceived Stress Questionnaire” (PSQ) an einer deutschen Stichprobe. Diagnostica.

[45] Kroenke K, Spitzer RL, Williams JBW, Löwe B (2009). An Ultra-Brief Screening Scale for Anxiety and Depression: The PHQ–4. Psychosomatics.

[46] Sijtsma K (2009). On the Use, the Misuse, and the Very Limited Usefulness of Cronbach’s Alpha. Psychometrika.

[47] Cronbach LJ (2004). My current thoughts on coefficient alpha and successor procedures. Educ Psychol Meas.

[48] Zinbarg RE, Revelle W, Yovel I, Li W (2005). Cronbach’s alpha, Revelle’s beta, and McDonald’s (omega H): Their relations with each other and two alternative conceptualizations of reliability. Psychometrika.

[49] Dunn TJ, Baguley T, Brunsden V (2014). From alpha to omega: a practical solution to the pervasive problem of internal consistency estimation. Br J Psychol.

[50] Schwarzer R, Jerusalem M (1999). Skalen zur Erfassung von Lehrer- und Schülermerkmalen. Dokumentation der psychometrischen Verfahren im Rahmen der wissenschaftlichen Begleitung des Modellversuchs Selbstwirksame Schulen.

[51] Scheier MF, Carver CS (1985). Optimism, coping, and health: assessment and implications of generalized outcome expectancies. Health Psychol.

[52] Zirke N, Schmid G, Mazurek B, Klapp BF, Rauchfuss M (2007). Antonovsky’s Sense of Coherence in psychosomatic patients - a contribution to construct validation. Psychosoc Med.

[53] Ahnis A, Riedl A, Figura A, Steinhagen-Thiessen E, Liebl ME, Klapp BF (2012). Psychological and sociodemographic predictors of premature discontinuation of a 1-year multimodal outpatient weight-reduction program: an attrition analysis. Patient Prefer Adherence.

[54] Mache S, Vitzthum K, Klapp BF, Danzer G (2014). Surgeons’ work engagement: influencing factors and relations to job and life satisfaction. Surgeon.

[55] Kocalevent R-D, Mierke A, Brähler E, Klapp BF, Kemper CJ, Brähler E, Zenger M (2014). BRCS - Brief Resilient Coping Scale. Psychologische und sozialwissenschaftliche Kurzskalen Standardisierte Erhebungsinstrumente für Wissenschaft und Praxis. Volume 1.

[56] Ahern NR, Kiehl EM, Sole ML, Byers J (2006). A review of instruments measuring resilience. Issues Compr Pediatr Nurs.

[57] Howe A, Smajdor A, Stockl A (2012). Towards an understanding of resilience and its relevance to medical training. Med Educ.

[58] Tempski P, Martins MA, Paro HB (2012). Teaching and learning resilience: a new agenda in medical education. Med Educ.

[59] Epstein RM, Krasner MS (2013). Physician resilience: what it means, why it matters, and how to promote it. Acad Med.

[60] Fertleman C, Carroll W (2013). Protecting students and promoting resilience. BMJ.

[61] Limonero JT, Tomas-Sabado J, Gomez-Romero MJ, Mate-Mendez J, Sinclair VG, Wallston KA, Gomez-Benito J (2014). Evidence for validity of the brief resilient coping scale in a young Spanish sample. Span J Psychol.

[62] Montero-Marin J, Piva Demarzo MM, Pereira JP, Olea M, Garcia-Campayo J (2014). Reassessment of the psychometric characteristics and factor structure of the ‘Perceived Stress Questionnaire’ (PSQ): analysis in a sample of dental students. PLoS One.

[63] Largo-Wight E, Peterson PM, Chen WW (2005). Perceived problem solving, stress, and health among college students. Am J Health Behav.

[64] Fliege H, Rose M, Arck P, Walter OB, Kocalevent R-D, Weber C, Klapp BF (2005). The Perceived Stress Questionnaire (PSQ) reconsidered: validation and reference values from different clinical and healthy adult samples. Psychosom Med.

[65] Spitzer RL, Williams JB, Kroenke K, Linzer M, De Gruy FV, Hahn SR, Brody D, Johnson JG (1994). Utility of a new procedure for diagnosing mental disorders in primary care. The PRIME-MD 1000 study. JAMA.

[66] Lowe B, Wahl I, Rose M, Spitzer C, Glaesmer H, Wingenfeld K, Schneider A, Brahler E (2010). A 4-item measure of depression and anxiety: validation and standardization of the Patient Health Questionnaire-4 (PHQ-4) in the general population. J Affect Disord.

[67] Downs N, Feng W, Kirby B, McGuire T, Moutier C, Norcross W, Norman M, Young I, Zisook S (2014). Listening to Depression and Suicide Risk in Medical Students: the Healer Education Assessment and Referral (HEAR) Program. Acad Psychiatry.

[68] Yoon S, Lee Y, Han C, Pae C-U, Yoon H-K, Patkar AA, Steffens DC, Kim Y-K (2014). Usefulness of the Patient Health Questionnaire-9 for Korean Medical Students. Acad Psychiatry.

[69] Moutier C, Norcross W, Jong P, Norman M, Kirby B, McGuire T, Zisook S (2012). The suicide prevention and depression awareness program at the University of California, San Diego School of Medicine. Acad Med.

[70] Zisook S, Downs N, Moutier C, Clayton P (2012). College students and suicide risk: prevention and the role of academic psychiatry. Acad Psychiatry.

[71] Tanaka JS, Bollen KA (1993). Multifaceted conceptions of fit in structural equation models. Testing structural equation models.

[72] Sharma S, Mukherjee S, Kumar A, Dillon WR (2005). A simulation study to investigate the use of cutoff values for assessing model fit in covariance structure models. J Bus Res.

[73] Hooper D, Coughlan J, Mullen MR (2008). Structural Equation Modelling: Guidelines for Determing Model Fit. Electron J Bus Res Methods.

[74] Kriston L, Gunzler C, Harms A, Berner M (2008). Confirmatory factor analysis of the German version of the international index of erectile function (IIEF): a comparison of four models. J Sex Med.

[75] Kocalevent RD, Hinz A, Brahler E, Klapp BF (2011). Regional and individual factors of stress experience in Germany: results of a representative survey with the perceived stress questionnaire (PSQ). Gesundheitswesen.

[76] Bergdahl J, Bergdahl M (2002). Perceived stress in adults: prevalence and association of depression, anxiety and medication in a Swedish population. Stress and Health.

[77] Koenig TW, Parrish SK, Terregino CA, Williams JP, Dunleavy DM, Volsch JM (2013). Core personal competencies important to entering students’ success in medical school: what are they and how could they be assessed early in the admission process?. Acad Med.

[78] Dyrbye LN, Power DV, Massie FS, Eacker A, Harper W, Thomas MR, Szydlo DW, Sloan JA, Shanafelt TD (2010). Factors associated with resilience to and recovery from burnout: a prospective, multi-institutional study of US medical students. Med Educ.

[79] Schermelleh-Engel K, Moosbrugger H, Muller H (2003). Evaluating the Fit of Structural Equation Models: Tests of Significance and Descriptive Goodness-of-Fit Measures. Meth Psychol Res.

[80] Stewart SM, Lam TH, Betson CL, Wong CM, Wong AM (1999). A prospective analysis of stress and academic performance in the first two years of medical school. Med Educ.

[81] Backovic DV, Zivojinovic JI, Maksimovic J, Maksimovic M (2012). Gender differences in academic stress and burnout among medical students in final years of education. Psychiatr Danub.

[82] Wild K, Scholz M, Ropohl A, Brauer L, Paulsen F, Burger PH (2014). Strategies against burnout and anxiety in medical education--implementation and evaluation of a new course on relaxation techniques (Relacs) for medical students. PLoS One.

[83] Deckro GR, Ballinger KM, Hoyt M, Wilcher M, Dusek J, Myers P, Greenberg B, Rosenthal DS, Benson H (2002). The evaluation of a mind/body intervention to reduce psychological distress and perceived stress in college students. J Am Coll Health.

[84] Liaison Committee on Medical Education: Functions and Structure of a Medical School. Standards for Accreditation of Medical Education Programs Leading to the MD Degree. 2014: 48. [http://lcme.org/publications/] Access Date: 26 Sept 2015

[85] General Medical Council: Supporting medical students with mental health conditions. 2015: 76 [http://www.gmc-uk.org/education/undergraduate/23289.asp] Access Date: 26 Aug 2016

